# Evaluation of Serum and Plasma Interleukin-6 Levels in Obstructive Sleep Apnea Syndrome: A Meta-Analysis and Meta-Regression

**DOI:** 10.3389/fimmu.2020.01343

**Published:** 2020-07-21

**Authors:** Mohammad Moslem Imani, Masoud Sadeghi, Habibolah Khazaie, Mostafa Emami, Dena Sadeghi Bahmani, Serge Brand

**Affiliations:** ^1^Department of Orthodontics, Kermanshah University of Medical Sciences, Kermanshah, Iran; ^2^Medical Biology Research Center, Kermanshah University of Medical Sciences, Kermanshah, Iran; ^3^Sleep Disorders Research Center, Kermanshah University of Medical Sciences, Kermanshah, Iran; ^4^Students Research Committee, Kermanshah University of Medical Sciences, Kermanshah, Iran; ^5^Center for Affective, Stress and Sleep Disorders, Psychiatric Clinics, University of Basel, Basel, Switzerland; ^6^Substance Abuse Prevention Research Center, Kermanshah University of Medical Sciences, Kermanshah, Iran; ^7^Departments of Physical Therapy, University of Alabama at Birmingham, Birmingham, AL, United States; ^8^Division of Sport Science and Psychosocial Health, Department of Sport, Exercise and Health, University of Basel, Basel, Switzerland; ^9^School of Medicine, Tehran University of Medical Sciences, Tehran, Iran

**Keywords:** obstructive sleep apnea syndrome, interleukin-6, serum, plasma, meta-analysis, pediatric and adult individuals

## Abstract

Obstructive sleep apnea syndrome (OSAS) is considered a low-grade chronic inflammatory disease. Interleukin-6 (IL-6) is one of the most significant inflammatory markers and an excellent proxy for the inflammatory/immune system. The present meta-analysis and meta-regression aimed at comparing plasma and serum levels of IL-6 between individuals (children and adults) with OSAS and healthy controls. Four databases, PubMed/Medline, Scopus, Cochrane Library, and Web of Science, were comprehensively searched to retrieve articles published up to December, 2019, with no further restrictions. RevMan 5.3 software was used to calculate the crude mean difference (MD) and 95% confidence interval (CI). The results of funnel plots and meta-regression were analyzed by the CMA 2.0 software. Sixty-three studies (57 with adults; six with children) were included in the present meta-analysis. For adults, 37 studies reported significantly higher serum IL-6 levels and 20 reported significantly higher plasma IL-6 levels for those with OSAS than for healthy controls [pooled MD of 2.89 pg/ml (*P* < 0.00001) and pooled MD of 2.89 pg/ml (*P* < 0.00001), respectively]. The pooled analysis of serum and plasma IL-6 levels in children with OSAS compared with controls revealed that only the MD of plasma IL-6 levels was significant (MD = 0.84 pg/ml, *P* = 0.004). Results of the meta-regression showed that greater age was associated with higher serum IL-6 levels. Egger's test revealed a publication bias across the studies for serum and plasma IL-6 levels (*P* = 0.00044 and *P* = 0.01445, respectively). In summary, the meta-analysis and meta-regression confirmed that, compared to healthy controls, individuals with OSAS (children and adults) had higher serum/plasma IL-6 levels.

## Introduction

Obstructive sleep apnea syndrome (OSAS) is a complex and multifactorial disease that includes upper airway obstruction, chronic intermittent hypoxia, and sleep fragmentation (1). This disease is associated with insulin resistance (2), obesity (3), and metabolic syndrome (4), and is a major risk factor for type II diabetes (5). OSAS is also associated with cardiovascular disease (6). One study found that the prevalence rate for moderate to severe OSAS was 23.4% in female individuals, 49.7% in male individuals, and 70% in individuals with obesity (7). Additionally, OSAS adds to the development of nonalcoholic fatty liver disease (8).

In both children and adults with OSAS, the upregulation has been observed of prototypic inflammatory cytokines such as interleukin-6 (IL-6), and this in turn increased the nuclear factor κB (NFκB) pathway activation (9). For these reasons, OSAS is considered a low-grade chronic inflammatory disease (10). Repetitive hypoxia and reoxygenation during OSAS lead to and trigger anti-inflammatory cascades (11). Inflammatory markers such as IL-6 trigger and enhance vascular inflammation (12) and contribute to OSAS-related cardiovascular morbidity (13). Indeed, IL-6 is a multifunctional cytokine with several biological activities such as the proliferation of T lymphocytes, the differentiation of B lymphocytes, and the stimulation of immunoglobulin secretion (14). Remarkably, along with other bone resorption agents, IL-6 activates bone resorption (15).

Two previous meta-analyses (9, 16) have examined blood IL-6 levels in individuals with OSAS and combined the results of serum and plasma blood IL-6 levels. Their results illustrated an elevated level of this cytokine in individuals with OSAS when compared to controls. In these meta-analyses with 35 studies in 2015 (16) and 15 studies in 2013 (9), the standard mean difference (MD) was used to analyze the data while there were no subgroup analyses based on ethnicity or number of participants. In addition, the meta-regressions performed in these two analyses (9, 16) evaluated the correlations between age, body mass index (BMI), apnea–hypopnea index (AHI) and IL-6 levels, while the more recent of the two (16) just included ethnicity in a subgroup analysis, but exclusively in the meta-regression. Additionally, the earlier meta-analysis (9) did not include children. To address the limitations of these previous meta-analyses, we separately analyzed the results for plasma and serum levels of IL-6 in adults and children with OSAS across 63 studies using MD. We also examined the correlations of age, BMI, AHI, publication year, and number of participants with IL-6 level based on a meta-regression. Last, unlike the two previous meta-analyses, we ran subgroup analyses for ethnicity, number of participants, BMI, age, and AHI.

## Materials and Methods

The review process followed the guidelines in the Preferred Reporting Items for Systematic Reviews and Meta-Analyses (PRISMA) statement (17).

### Search Strategy

Four databases, PubMed/Medline, Scopus, Cochrane Library, and Web of Science, were comprehensively searched by one author (MS) to retrieve articles published up to 1st December, 2019, with no restrictions. The searched terms were (“sleep apnea” or “obstructive sleep apnea” or “obstructive sleep apnea syndrome” or “OSA” or “OSAS”) and (“interleukin-6” or “IL-6” or “interleukin 6”) and (“plasma” or “blood” or “serum”). We also manually searched for references (original and review articles) related to these topics.

### Eligibility Criteria

Inclusion criteria were the following: (1) studies assessing the association between plasma or serum IL-6 levels and OSAS with case–control design without age, sex, or BMI restrictions; (2) OSAS was defined as AHI > 5 events/h in adults, and AHI > 1 events/h in children; (3) OSAS was diagnosed with polysomnography; (4) individuals with OSAS and controls had no other systematic diseases such as diabetes, neurological disorders such as multiple sclerosis, or neurodegenerative disorders such as Alzheimer's; (5) studies reporting pretreatment morning serum and/or plasma levels of IL-6 (around 6–10 am); (6) studies reporting sufficient data to calculate the MD and 95% confidence interval (CI) in individuals with OSAS and controls; and (7) studies with more than 10 cases included as individuals with OSAS and controls.

Exclusion criteria were the following: (1) studies with irrelevant or insufficient data or without clinical data; (2) review articles, conference papers, and book chapters; (3) studies without a control group; (4) studies reporting controls with AHI > 5 events/h in adults, and AHI > 1 events/h in children; and (5) studies reporting data overlapping with other studies.

### Study Selection

Two authors (MMI and ME) independently read the titles and abstracts of the retrieved studies. Then, these two authors selected the relevant studies, while another author (MS) retrieved the full texts of the articles and excluded several full texts for the following reasons: two were meta-analyses; five were reviews; two were letter to editor studies; 25 had no control condition, or the control group was selected as AHI > 5 events/h in adults and AHI > 1 events/h in children; one had <10 cases included in both groups; six had no relevant data; one had duplicated data with another study; two did not report baseline IL-6 levels; one reported polymorphisms of IL-6; one reported high sensitivity IL-6 levels; three did not report morning level of IL-6; and one reported the level of IL-6 in exhaled breath condensate. Where two studies had overlapping data, we selected the study with the more recent publication year.

### Data Extraction

Two authors (SB and MS) independently extracted the data from each study included in the meta-analysis. If there was a disagreement between the two authors, a third author (MMI) helped to reach a final decision. The data extracted for the meta-analysis included basic information including the first author, publication year, country of study, ethnicity of participants, age, BMI, and AHI of both groups, and mean and standard deviation (SD) of IL-6 levels in plasma and serum. Since some studies considered patients with mild OSAS (AHI, 1–5 events/h) as a control group condition, and since we had used this protocol for other studies, reporting the quality of studies as for usual case–control studies did not seem to be reasonable.

### Quality Assessment

One author (MS) assessed the quality of the studies included in the meta-analysis using the Newcastle–Ottawa Scale (NOS); the total possible score for each study was 9 (18).

### Statistical Analyses

The data were analyzed by one author. Review Manager 5.3 software (RevMan 5.3) was used to calculate the crude MD and 95% CI, which evaluated the significance of the pooled MD by *Z* test. Heterogeneity was assessed across the studies using both Cochran *Q* (19) and *I*^2^ metrics with scores ranging from 0 to 100% (20). In addition, in case of values of *P*_heterogeneity_ (*P*_h_ < 0.1) and *I*^2^ > 50%, this indicated a significant heterogeneity, and in this case, analysis of the random effect model was performed to evaluate the pooled MD and 95% CI values. Otherwise, we used the analysis by the fixed effect model.

The results of Begg's and Egger's tests were analyzed by the Comprehensive Meta-Analysis version 2.0 software (CMA 2.0). Begg's funnel plot shows the standard error (SE) of the log (MD), and the precision of each study is plotted against its log (MD) (21). Egger's test shows the linear regression between the precision of the studies and the standardized effect (22). Subgroup analyses were performed based on ethnicity, AHI, BMI, and sample size. Sensitivity analyses, namely, the “cumulative analysis” and “one study removed,” were used to estimate the consistency/stability of the results. A *P* value (two-tailed) of < 0.05 was taken to indicate a significant difference. Meta-regression is a quantitative method used in meta-analyses to estimate the impact of moderator variables on study effect size. The trim-and-fill method was used to estimate potentially missing studies due to publication bias in the funnel plot and to adjust the overall effect estimate (23).

Some studies reported the values for IL-6 in standard errors (SE); SEs were transformed into standard deviations (SD), (SE = SD/√*N*; *N* = number of individuals). Some studies reported median and interquartile values, which were transformed into mean and SD (24). The IL-6 levels in the serum and plasma were reported in picograms per milliliter (pg/ml). Participants with BMI >30 kg/m^2^ were considered obese (25).

## Results

### Search Results

**Figure 1:**
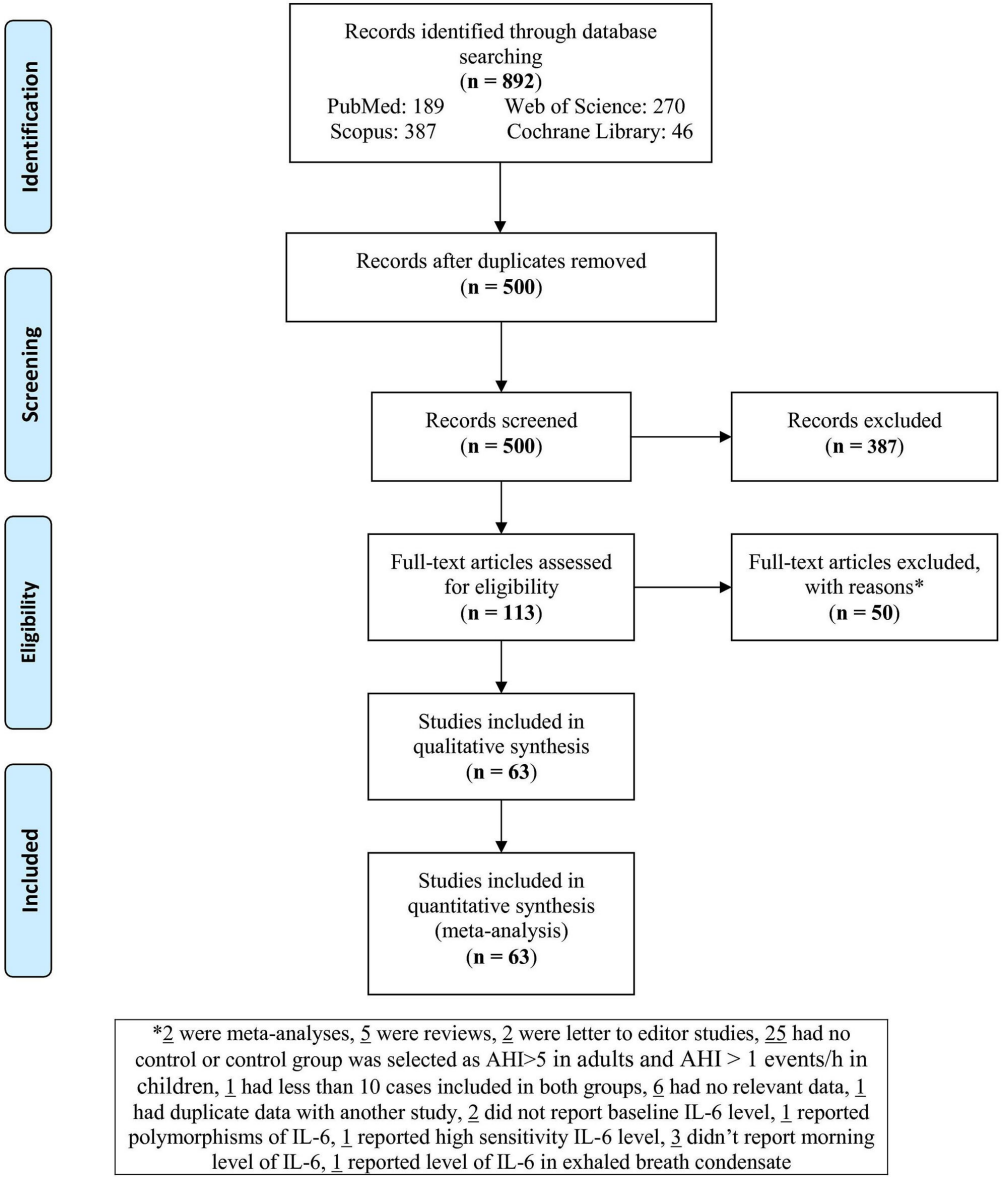
Flowchart of the study.

A total of 892 records were identified in the four electronic databases. Figure 1 sets out the literature selection of these studies. After excluding duplications and irrelevant records, 113 full-text articles met the eligibility criteria. After evaluating the full texts, 50 articles were excluded as mentioned in *Study Selection*. At the end, 63 studies were included in the present meta-analysis (12, 26–87).

### Basic Characteristics of the Studies

As shown in Table 1, of the 63 studies retained for analysis, 14 were conducted in China (27, 39, 46, 50, 51, 54, 55, 58, 65, 73, 77, 81, 82, 86), eight in the USA (26, 28, 34, 38, 56, 76, 78, 83), six in Greece (37, 43, 47, 49, 58, 64), six in Japan (29–31, 41, 42, 44), six in Turkey (12, 57, 62, 67, 68, 70), three in Italy (59, 63, 87), 3 in Brazil (45, 53, 72), three in Spain (36, 60, 71), two in Sweden (66, 84), two in India (79, 85), one in Ireland (32), one in Germany (35), one in Thailand (40), one in Finland (48), one in Canada (52), one in the UK (74), one in Croatia (80), one in Australia (33), one in Taiwan (69), and one in Belgium (75). With regard to ethnicity, 28 studies were performed with Caucasians (12, 32, 35–37, 43, 47–49, 57, 59–64, 66–68, 70, 71, 74, 75, 79, 80, 84, 85, 87), 22 with Asians (27, 29–31, 39–42, 44, 46, 50, 51, 54, 55, 58, 65, 69, 73, 77, 81, 82, 86), and 13 with mixed ethnicities (26, 28, 33, 34, 38, 45, 52, 53, 56, 72, 76, 78, 83). Of the 63 studies, 40 reported IL-6 levels in serum (12, 28, 30–34, 37, 39, 40, 44, 46, 47, 49–57, 62, 63, 65–68, 70, 72–77, 79, 81, 82, 85, 87), and 23 reported IL-6 levels in plasma (26, 27, 29, 35, 36, 38, 41–43, 45, 48, 55, 58–61, 64, 69, 71, 78, 80, 83, 84). Six studies included children (33, 38, 39, 60, 69, 75). Other characteristics of individuals with OSAS and controls (number of cases, age, BMI, and AHI) are shown in Figure 1.

**Table 1:** Characteristics of studies included in the meta-analysis (*n* = 63).

**First author, year**	**Country**	**Ethnicity**	**No. of OSAS/control**	**OSAS patients**			**Controls**			**Sample**
				**Age (years)**	**BMI (kg/m^**2**^)**	**AHI (events/h)**	**Age (years)**	**BMI (kg/m^**2**^)**	**AHI (events/h)**	
**ADULTS**										
Vgontzas, 1997 (26)	USA	Mixed	12/10	40.9 ± 2.2	40.5 ± 3.2	63.7 ± 10.3	24.1 ± 0.8	24.6 ± 0.7	0 ± 0	Plasma
Huiguo, 2000 (27)	China	Asian	22/16	47.4 ± 13.6	27.6 ± 3.3	44.0 ± 21.0	47.6 ± 14.7	23.1 ± 3	4.29 ± 2.16	Plasma
Roytblat, 2000 (28)	USA	Mixed	11/12	39.5 ± 5.0	38.3 ± 8.0	≥5	32.8 ± 8.2	21.0 ± 5.3	<5	Serum
Teramoto, 2003 (29)	Japan	Asian	40/40	Adult	NR	≥5	Adult	NR	<5	Plasma
Yokoe, 2003 (30)	Japan	Asian	26/14	52.5 ± 5.7	28.4 ± 3.5	33.7 ± 24.5	48.8 ± 3.0	27.6 ± 0.5	2.8 ± 0.2	Serum
Ciftci, 2004 (12)	Turkey	Caucasian	43/22	49.6 ± 9.1	31.9 ± 4.1	33.2 ± 5.0	47.2 ± 10.3	31.03 ± 3.1	1.55 ± 0.96	Serum
Imagawa, 2004 (31)	Japan	Asian	117/46	Adult	27.7 ± 4.4	≥5	Adult	22.9 ± 2.9	<5	Serum
Ryan, 2006 (32)	Ireland	Caucasian	66/30	42.5 ± 8.5	32.5 ± 4.8	35.0 ± 13.9	41 ± 8	30.7 ± 3.1	1.2 ± 1.0	Serum
de la Peña Bravo, 2007 (34)	USA	Mixed	50/20	51.7 ± 1.9	33.2 ± 1.7	51.3 ± 4.2	47.4 ± 1.2	28.4 ± 0.6	2.5 ± 0.5	Serum
Harsch, 2007 ((35)	Germany	Caucasian	19/20	59.0 ± 2.0	34.4 ± 1.4	≥5	53.0 ± 2.0	31.6 ± 1.0	<5	Plasma
Arias, 2008 (36)	Spain	Caucasian	30/15	52.0 ± 13.0	30.5 ± 4.0	43.8 ± 27.0	48.0 ± 10.0	28.7 ± 4.7	3.7 ± 3.3	Plasma
Constantinidis, 2008 (37)	Greece	Caucasian	24/27	45.1 ± 8.2	≥25	23.3 ± 3.6	45.1 ± 8.2	≥25	3.5 ± 0.4	Serum
Li, 2008 (40)	Thailand	Asian	68/22	45.5 ± 11.3	27.7 ± 4.6	31.4 ± 28.6	43 ± 9	23.3 ± 2.0	2.9 ± 1.3	Serum
Takahashi, 2008 (41)	Japan	Asian	41/12	49.8 ± 10	29.4 ± 4.2	≥5	46.7 ± 11.2	25.7 ± 4.1	<5	Plasma
Tomiyama, 2008 (42)	Japan	Asian	50/15	51.4 ± 13.0	26.9 ± 4.2	42.7 ± 27.9	53.0 ± 10.0	24.3 ± 2.5	<5	Plasma
Vgontzas, 2008 (43)	Greece	Caucasian	16/28	48.1 ± 5.6	37.5 ± 4.7	53.3 ± 7.0	48.1 ± 10.6	30.8 ± 5.2	2.1 ± 0.8	Plasma
Yamamoto, 2008 (44)	Japan	Asian	31/10	49.0 ± 2.0	28.0 ± 1.0	≥5	46.0 ± 6.0	27.0 ± 2.0	<5	Serum
Carneiro, 2009 (45)	Brazil	Mixed	16/13	40.1 ± 2.8	46.9 ± 2.0	65.7 ± 9.9	38.8 ± 3.3	42.8 ± 1.3	3.2 ± 0.5	Plasma
Li, 2009 (46)	China	Asian	68/22	45.3 ± 11.1	20.7 ± 10.1	38.9 ± 26.5	43.0 ± 93.0	23.3 ± 2.0	2.9 ± 1.3	Serum
Thomopoulos, 2009 (47)	Greece	Caucasian	62/70	48.1 ± 7.6	31.9 ± 4.9	31.6 ± 2.0	48.1 ± 3.9	32.1 ± 3.0	0.4 ± 4.0	Serum
Sahlman, 2010 (48)	Finland	Caucasian	84/40	50.4 ± 9.3	32.5 ± 3.3	9.6 ± 2.9	45.6 ± 11.5	31.5 ± 3.5	1.9 ± 1.4	Plasma
Steiropoulos, 2010 (49)	Greece	Caucasian	38/23	45.5 ± 10.5	36.4 ± 7.4	61.0 ± 27.0	43.7 ± 6.7	34.5 ± 3.7	5.3 ± 3.2	Serum
Ye, 2010 (50)	China	Asian	127/52	45.3 ± 11.4	26.2 ± 3.7	36.5 ± 25.7	45.0 ± 10.0	26.0 ± 3.2	2.0 ± 1.4	Serum
Liu, 2011(51)	China	Asian	78/20	45.2 ± 7.3	27.9 ± 3.1	46.3 ± 9.2	43.5 ± 8.3	26.1 ± 2.4	<5	Serum
Fornadi, 2012 (52)	Canada	Mixed	25/75	54.0 ± 12.0	29.0 ± 5.0	≥5	50.0 ± 13.0	26.0 ± 5.0	<5	Serum
Medeiros, 2012 (53)	Brazil	Mixed	50/15	62.3 ± 7.8	25.5 ± 4.0	≥5	62.50 ± 8.4	25.8 ± 4.0	<5	Serum
Qian, 2012 (54)	China	Asian	70/40	45.8 ± 8.2	28.9 ± 2.3	≥5	46.3 ± 8.1	24.1 ± 2.3	<5	Serum
Ye, 2012 (55)	China	Asian	44/20	46.43 ± 18.22	28.2 ± 5.34	40.41 ± 20.68	45.80 ± 23.01	26.90 ± 4.25	2.26 ± 1.30	Plasma
Hargens, 2013 (56)	USA	Mixed	12/33	22.8 ± 0.8	32.4 ± 1.0	25.4 ± 5.4	21.9 ± 0.6	29.3 ± 0.5	2.1 ± 0.3	Serum
Kurt, 2013 (57)	Turkey	Caucasian	48/37	48.3 ± 12.3	NR	≥5	43.1 ± 14.1	NR	<5	Serum
Yang, 2013 (58)	China	Asian	50/25	53.5 ± 6.9	27.4 ± 2.9	24.5 ± 15.9	53.0 ± 7.0	26.27 ± 1.9	3.0 ± 1.0	Plasma
Ciccone, 2014 (59)	Italy	Caucasian	80/40	52.8 ± 10.6	28.6 ± 3.0	33.9 ± 21	52.3 ± 10.5	28.2 ± 2.7	2.1 ± 1.1	Plasma
Kritikou, 2014 (61)	Greece	Caucasian	38/39	55.7 ± 6.6	28.7 ± 3.3	37.5 ± 21.0	53.8 ± 6.2	27.3 ± 3.5	2.3 ± 1.9	Plasma
Unuvar Dogan, 2014 (62)	Turkey	Caucasian	33/24	45.3 ± 8.5	31.0 ± 1.7	47.2 ± 23.2	40.5 ± 9.5	30.7 ± 1.5	3.6 ± 1.8	Serum
De, 2015 (63)	Italy	Caucasian	26/24	41.8 ± 7.4	33.0 ± 5.2	26.15 ± 12.1	43.7 ± 8.2	30.8 ± 4.3	1.65 ± 0.9	Serum
Gaines, 2015 (64)	Greece	Caucasian	82/38	54.9 ± 5.8	29.2 ± 3.3	22.6 ± 15.0	54.2 ± 5.8	27.6 ± 3.7	2.0 ± 15.0	Plasma
Hui, 2015 (65)	China	Asian	35/20	47.6 ± 6.6	27.3 ± 4.4	≥5	25.1 ± 5.3	40.5 ± 4.9	<5	Serum
Ulasli, 2015 (67)	Turkey	Caucasian	62/20	51.7 ± 10.2	32.4 ± 5.6	26.5 ± 18.5	45.3 ± 14	30.4 ± 8	2.2 ± 0.93	Serum
Thunstrom, 2015 (66)	Sweden	Caucasian	344/95	61.9 ± 9.2	28.9 ± 4.0	29.7 ± 14.6	61.4 ± 9.5	25.2 ± 2.5	3.1 ± 1.3	Serum
Dogan, 2016 (68)	Turkey	Caucasian	39/12	40.7 ± 10.75	29.26 ± 4.12	≥5	40.0 ± 11.7	27.29 ± 2.93	<5	Serum
Nizam, 2016 (70)	Turkey	Caucasian	39/13	47.3 ± 10.4	33.2 ± 56.4	45.6 ± 20.7	43.23 ± 9.08	31.71 ± 4.56	2.64 ± 1.82	Serum
Vicente, 2016 (71)	Spain	Caucasian	89/26	45.33 ± 14.81	30.03 ± 5.04	28 ± 23.70	45 ± 11.11	28.7 ± 4.37	1.9 ± 2.7	Plasma
Hirotsu, 2017 (72)	Brazil	Mixed	339/682	50.8 ± 13.2	29.6 ± 5.8	19.3 ± 9.44	38.2 ± 12.7	25.4 ± 3.8	2.5 ± 10.4	Serum
Kong, 2017 (73)	China	Asian	82/30	45.6 ± 8.8	27.7 ± 4.6	≥5	45.7 ± 6.6	27.6 ± 3.6	<5	Serum
Thorn, 2017 (74)	UK	Caucasian	16/14	59 ± 13	32.7 ± 4.0	30 ± 18	58 ± 7	30.6 ± 2.7	<5	Serum
Weingarten, 2017 (76)	USA	Mixed	11/8	38.7 ± 10.5	47.3 ± 14.8	25.1 ± 17.8	33.6 ± 5.1	25.5 ± 10.9	1.8 ± 1.0	Serum
Zhang, 2017 (77)	China	Asian	50/52	79.4	NR	≥5	77.1	NR	<5	Serum
Al-Terki, 2018 (78)	USA	Mixed	52/22	40.3 ± 1.6	29.5 ± 0.7	22.9 ± 17.9	39.6 ± 2.2	28.5 ± 0.9	2.5 ± 1.6	Plasma
Bhatt, 2018 (79)	India	Caucasian	171/69	44.6 ± 9.1	33.1 ± 7.6	≥5	40.0 ± 9.8	30.1 ± 8.4	<5	Serum
Bozic, 2018 (80)	Croatia	Caucasian	50/25	53.0 ± 11.9	28.9 ± 2.7	35.0 ± 11.0	52.5 ± 10.2	27.8 ± 2.2	<5	Plasma
Kong, 2018 (81)	China	Asian	50/40	54.34 ± 14.38	26.86 ± 3.12	37.34 ± 19.02	50.42 ± 8.35	22.26 ± 3.54	3.31 ± 1.09	Serum
Lu, 2018 (82)	China	Asian	35/22	48.63 ± 1.80	28.21 ± 0.51	≥5	49.04 ± 2.27	26.01 ± 0.65	<5	Serum
Motamedi, 2018 (83)	USA	Mixed	50/24	34.9 ± 8.0	30.8 ± 4.2	18.4 ± 13.1	30.9 ± 7.77	28.6 ± 3.81	2.12 ± 1.3	Plasma
Sundbom, 2018 (84)	Sweden	Caucasian	109/224	55.6 ± 8.8	28.2 ± 4.8	25.4 ± 2.1	47.7 ± 11.3	25.4 ± 4.1	3.7 ± 0.6	Plasma
Bhatt, 2019 (85)	India	Caucasian	47/25	44.2 ± 9.1	32.5 ± 6.9	13.5 ± 6.4	28.5 ± 8.6	41 ± 8.5	2.3 ± 1.1	Serum
Tang, 2019 (86)	China	Asian	120/127	48.88 ± 9.76	26.86 ± 3.12	39.00 ± 18.38	47.37 ± 9.12	22.46 ± 3.29	3.31 ± 1.09	Serum
Galati, 2020 (87)	Italy	Caucasian	45/30	53.9 ± 11.6	28 ± 2.2	≥5	55 ± 5.8	26.3 ± 1.8	<5	Serum
**CHILDREN**										
Tam, 2006 (33)	Australia	Mixed	44/69	7.3 ± 3.7	19.4 ± 5.5	5.3 ± 6.5	7.6 ± 4.0	17.9 ± 3.9	0 ± 0	Serum
Gozal, 2008 (38)	USA	Mixed	20/20	6.5 ± 0.6	17.3 ± 0.6	≥5	6.4 ± 0.7	17.1 ± 0.5	0 ± 0	Plasma
Li, 2008 (39)	China	Asian	47/95	11.1 ± 1.27	NR	14.1 ± 8.0	10.7 ± 1.3	NR	0.7 ± 0.6	Serum
Gileles-Hillel, 2014 (60)	Spain	Caucasian	75/129	10.4 ± 2.8	28.0 ± 4.6	14.2 ± 9.0	11.0 ± 2.4	27.9 ± 4.1	0.6 ± 0.6	Plasma
Huang, 2016 (69)	Taiwan	Asian	47/32	7.84 ± 0.56	16.95 ± 0.47	9.13 ± 1.67	7.02 ± 0.65	16.55 ± 0.58	0.37 ± 0.06	Plasma
Van Eyck, 2017 (75)	Belgium	Caucasian	53/111	11.5 ± 2.75	NR	20.53 ± 15.98	12 ± 2.75	NR	0.72 ± 0.47	Serum

*NR, not reported; OSAS, obstructive sleep apnea syndrome; AHI, apnea–hypopnea index; BMI, body mass index*.

### Meta-Analysis

#### Serum IL-6 Levels in Individuals With Obstructive Sleep Apnea Syndrome

**Figure 2:**
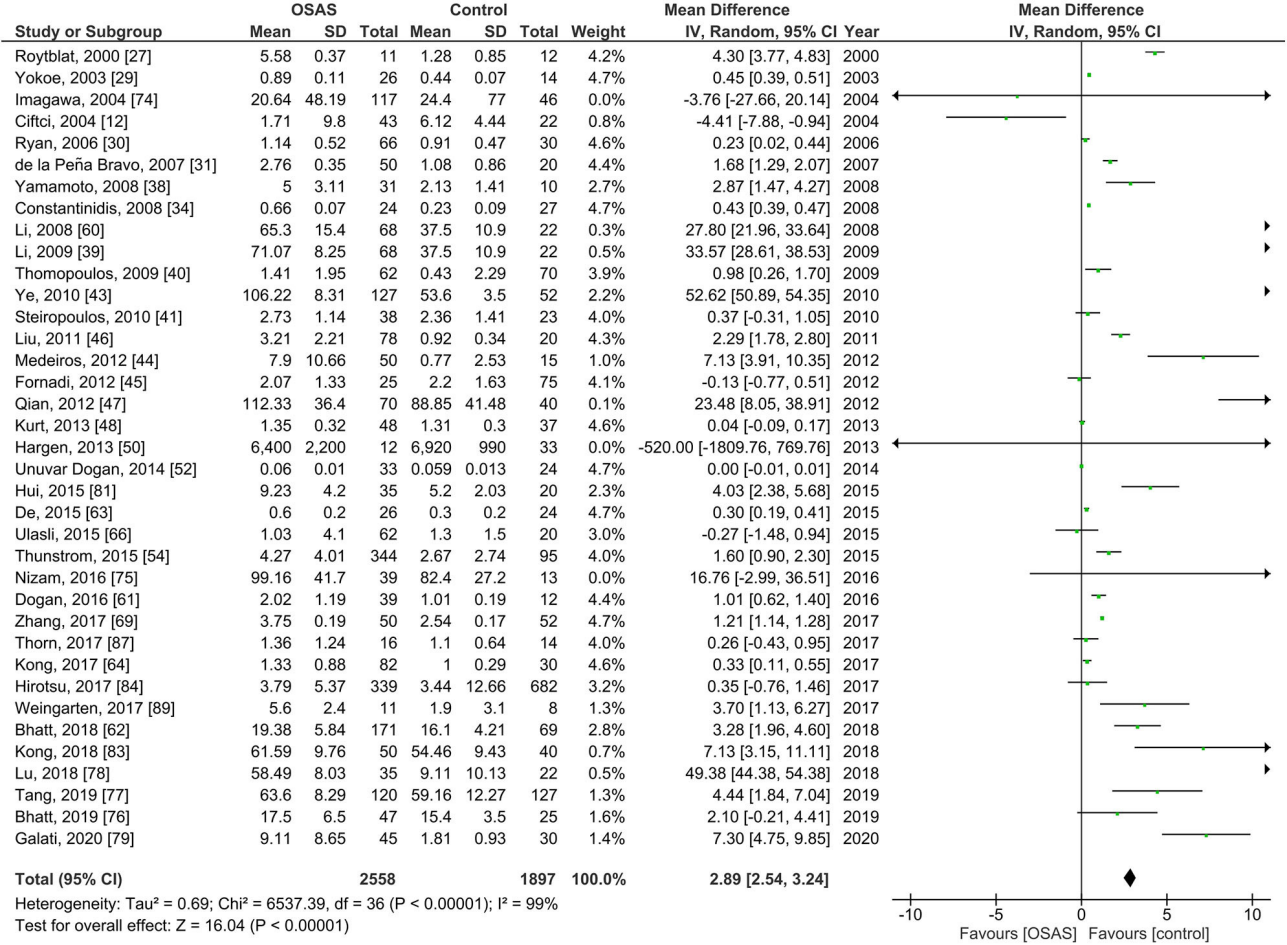
Forest plot of random-effects analysis of serum interleukin-6 levels in adults. The diamond shape indicates the pooled mean difference (MD). Each black box represents a point estimate of each study and also gives a representation of the study size (the bigger the box, the more participants in the study). A horizontal line representing the 95% confidence intervals (CIs) of the study result, with each end of the line representing the boundaries of CI. SD, standard deviation; OSAS, obstructive sleep apnea syndrome.

Thirty-nine studies of serum IL-6 levels in adults included 2,558 individuals with OSAS and 1,897 controls (Figure 2). The pooled analysis of individuals with OSAS compared to controls yielded an MD of 2.89 pg/ml [95% CI: 2.54, 3.23; *P* < 0.00001; *I*^2^ = 99% (*P*_h_ < 0.00001)]. Thus, serum levels of IL-6 were significantly higher in individuals with OSAS than in controls.

#### Plasma IL-6 Levels in Individuals With Obstructive Sleep Apnea Syndrome

**Figure 3:**
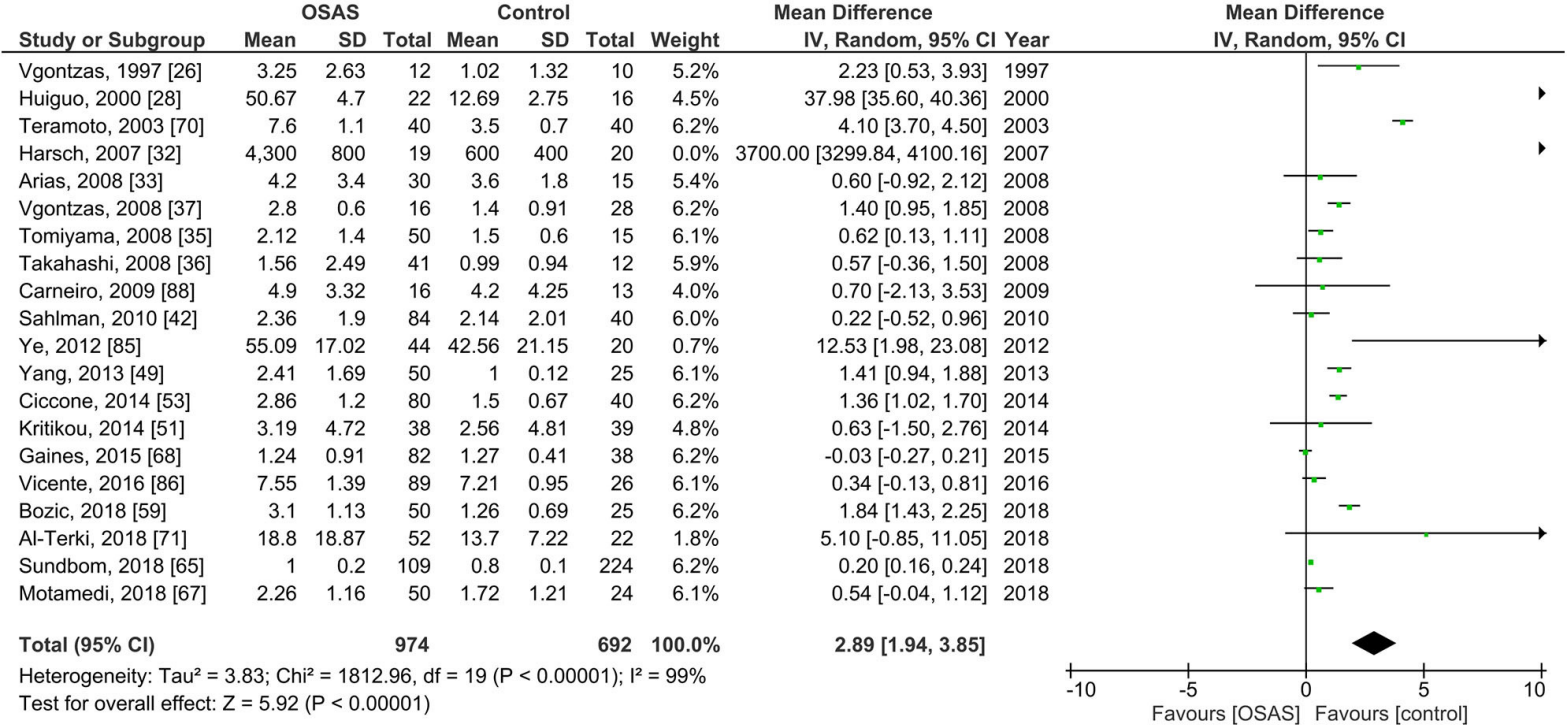
Forest plot of random-effects analysis of plasma interleukin-6 levels in adults. The diamond shape indicates the pooled mean difference (MD). Each black box represents a point estimate of each study and also gives a representation of the study size (the bigger the box, the more participants in the study). A horizontal line representing the 95% confidence intervals (CIs) of the study result, with each end of the line representing the boundaries of CI. SD, standard deviation; OSAS, obstructive sleep apnea syndrome.

Figure 3 shows the results of 22 studies reporting plasma IL-6 levels adults; plasma IL-6 levels of 974 individuals with OSAS were compared to the levels of 692 controls. The pooled analysis comparing these two groups showed that the MD was 2.89 pg/ml [95% CI: 1.94, 3.85; *P* < 0.00001; *I*^2^ = 99% (*P*_h_ < 0.00001)]. Thus, plasma IL-6 levels of individuals with OSAS were significantly higher than those of controls.

#### Serum IL-6 Levels in Children With Obstructive Sleep Apnea Syndrome

**Figure 4:**
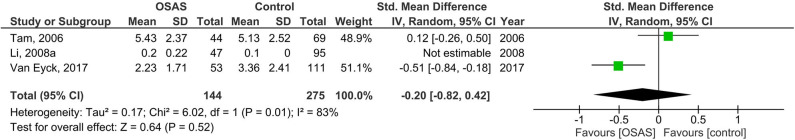
Forest plot of random-effects analysis of serum interleukin-6 levels in children. The diamond shape indicates the pooled mean difference (MD). Each black box represents a point estimate of each study and also gives a representation of the study size (the bigger the box, the more participants in the study). A horizontal line representing the 95% confidence intervals (CIs) of the study result, with each end of the line representing the boundaries of CI. SD, standard deviation; OSAS, obstructive sleep apnea syndrome.

Figure 4 illustrates the results of three studies on serum IL-6 levels of 144 children with OSAS compared to 274 controls. The pooled analysis of the comparison between these groups revealed that the MD was −0.20 pg/ml [95% CI: −0.82, 0.42; *P* = 0.52; *I*^2^ = 83% (*P*_h_ = 0.01)]. Thus, serum IL-6 levels did not significantly differ between children with OSAS and controls.

#### Plasma IL-6 Levels in Pediatric Participants With Obstructive Sleep Apnea Syndrome

**Figure 5:**
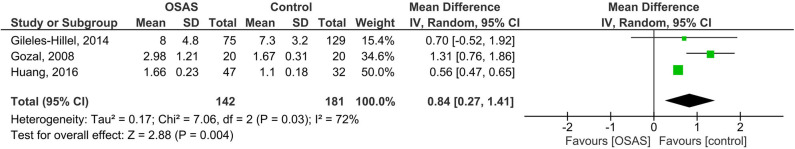
Forest plot of random-effects analysis of plasma interleukin-6 levels in children. The diamond shape indicates the pooled mean difference (MD). Each black box represents a point estimate of each study and also gives a representation of the study size (the bigger the box, the more participants in the study). A horizontal line representing the 95% confidence intervals (CIs) of the study result, with each end of the line representing the boundaries of CI. SD, standard deviation; OSAS, obstructive sleep apnea syndrome.

Five studies of plasma IL-6 levels in children included 142 children with OSAS and 181 controls (see Figure 5). The pooled analysis in this case revealed that the MD was 0.84 pg/ml [95% CI: 0.27, 1.41; *P* = 0.004; *I*^2^ = 72% (*P*_h_ = 0.03)]. The plasma IL-6 levels of children with OSAS were thus significantly higher than those of the controls.

### Subgroup Analysis of Serum IL-6 Levels

#### Ethnicity

Subgroup analyses of serum IL-6 levels in adults are reported in Table 2. The pooled analysis showed that for those with OSAS, serum IL-6 levels in Caucasian (MD = 0.51 pg/ml, *P* < 0.00001), Asian (MD = 11.52 pg/ml, *P* < 0.00001) and mixed (MD = 2.49 pg/ml, *P* = 0.004) ethnicities were significantly higher than the serum IL-6 levels of the respective controls.

**Table 2:** Subgroup analysis for serum and plasma levels of inteleukin-6 in adults.

**Subgroup analysis of serum level (*n*)**	**MD (95% CI), *P*-value, *I*^**2**^ (%), *P*_**h**_**	**Subgroup analysis of plasma level (*n*)**	**MD (95% CI), *P*-value, *I*^**2**^ (%), *P*_**h**_**
Overall (37)	**2.89 (2.54, 3.24)**, **<0.00001, 99**, **<0.00001**	Overall (20)	**2.89 (1.94, 3.85)**, **<0.00001, 99**, **<0.00001**
Ethnicity		Ethnicity	
Caucasian (16)	**0.51 (0.29, 0.73)**, **<0.00001, 97**, **<0.00001**	Caucasian (10)	0.75 (−0.06, 1.57), 0.07, 98,<0.00001
Asian (14)	**11.52 (10.20, 12.83)**, **<0.00001, 100**, **<0.00001**	Asian (6)	**8.93 (4.87, 12.99)**, ** <0.0001, 100**, **<0.00001**
Mixed (7)	**2.49 (0.80, 4.17), 0.004, 96**, **<0.00001**	Mixed (4)	**0.75 (0.21, 1.29), 0.006, 45, 0.14**
Mean BMI of OSAS patients, kg/m^2^		Mean BMI of OSAS patients, kg/m^2^	
>30 (15)	**1.07 (0.63, 1.50)**, **<0.00001, 97**, **<0.00001**	>30 (8)	0.96 (−1.16, 3.07), 0.38, 98, <0.00001
≤ 30 (19)	**10.59 (8.64, 12.53)**, **<0.00001, 100**, **<0.00001**	≤ 30 (11)	**4.20 (2.95, 5.46)**, **<0.00001, 99**, **<0.00001**
Mean BMI of controls, kg/m^2^		Mean BMI of controls, kg/m^2^	
>30 (12)	**0.47 (0.19, 0.76), 0.001, 89**, **<0.00001**	>30 (4)	1.69 (−4.61, 8.00), 0.60, 99, <0.00001
≤ 30 (22)	**9.55 (7.85, 11.25)**, **<0.00001, 100**, **<0.00001**	≤ 30 (15)	**3.05 (2.096, 4.01)**, **<0.00001, 99**, **<0.00001**
Total number of participants		Total number of participants	
>100 (9)	**9.52 (2.94, 16.11), 0.005, 100**, **<0.00001**	>100 (5)	0.42 (0.00, 0.84), 0.05, 92, <0.00001
≤ 100 (28)	**1.43 (1.14, 1.71)**, **<0.00001, 99**, **<0.00001**	≤ 100 (15)	**4.56 (2.51, 6.61)**, ** <0.0001, 99**, **<0.00001**
Mean AHI of OSAS patients, events/h		Mean AHI of OSAS patients, events/h	
>30 (14)	**6.13 (5.31, 6.95)**, **<0.00001, 100**, **<0.00001**	>30 (9)	**5.87 (3.37, 8.37)**, **<0.00001, 99**, **<0.00001**
≤ 30 (10)	**1.93 (1.20, 2.66)**, **<0.00001, 98**, ** <0.000101**	≤ 30 (7)	**0.43 (0.10, 0.75), 0.01, 82**, **<0.00001**

BMI, body mass index; CI, confidence interval; OSAS, obstructive sleep apnea syndrome; MD, mean difference; P_h_, P_heterogeneity_.

*Bold numbers show statistically significant value (P < 0.05)*.

#### Body Mass Index

Compared to healthy controls, the pooled MD of serum IL-6 levels of individuals with OSAS was significantly higher irrespective of their BMI: mean BMI > 30 (MD = 1.07 pg/ml, *P* < 0.00001); mean BMI ≤ 30 kg/m^2^ (MD = 10.59 pg/ml, *P* < 0.00001).

Compared to OSAS individuals with mean BMI > 30 kg/m^2^, OSAS individuals with BMI ≤ 30 kg/m^2^ had the pooled MD in serum IL-6 levels 9.9 times higher than controls.

Serum IL-6 levels were higher in participants with OSAS, irrespective of whether the BMI of controls was >30 (pooled MD of serum IL-6 levels was 0.47 pg/ml, *P* = 0.001), or ≤ 30 kg/m^2^ (pooled MD of serum IL-6 levels was 9.55 pg/ml, *P* < 0.00001). For participants with OSAS, serum IL-6 levels were 20.3 times higher in controls with a BMI <30 kg/m^2^ when compared to controls with a mean BMI > 30 kg/m^2^.

#### Sample Sizes

With regard to the number of participants in each study, those with more than 100 cases across OSAS and control groups had a pooled MD of serum IL-6 levels of 9.52 pg/ml (*P* = 0.005); the studies with ≤ 100 cases across the two groups had pooled MD of 1.43 pg/ml (*P* < 0.00001). In other words, serum IL-6 levels in studies with more than 100 cases were 6.6 times higher than the levels of studies including ≤ 100 cases across OSAS and control groups.

#### Apnea–Hypopnea Index

Studies with a mean AHI > 30 events/h in participants with OSAS showed that the pooled MD of their serum IL-6 levels was 6.13 pg/ml (*P* < 0.00001), compared to controls. Studies including participants with OSAS with a mean AHI ≤ 30 events/h showed that the pooled MD between OSAS individuals and controls was 1.93 pg/ml (*P* < 0.00001). Thus, serum IL-6 levels in studies with mean AHI > 30 events/h in participants with OSAS were 3.2 times higher than serum IL-6 levels in studies including OSAS patients with mean AHI ≤ 30 events/h.

### Subgroup Analysis of Plasma IL-6 Levels

#### Ethnicity

The subgroup analysis of the pooled MD for plasma IL-6 levels in Asian (MD = 8.93 pg/ml, *P* = 0.0001) and mixed ethnicity (MD = 0.75 pg/ml, *P* = 0.006) participants with OSAS showed significant differences with the respective controls. The result showed that plasma IL-6 levels of Asians were 11.9 times higher than the plasma IL-6 levels of those of mixed ethnicity with OSAS. Plasma IL-6 levels of Asians did not statistically significantly differ from the plasma level of a Caucasian ethnicity.

#### Body Mass Index

In studies including participants with a mean BMI ≤ 30 kg/m^2^, the pooled MD comparing plasma IL-6 levels in participants with OSAS with plasma IL-6 levels of controls was significant (MD = 4.20 pg/ml, *P* < 0.00001). The pooled MD was also significant for plasma IL-6 levels in studies including a mean BMI of controls ≤ 30 (MD = 3.05 pg/ml, *P* < 0.00001). In contrast, there was no significant difference between participants with or without OSAS when the BMI of OSAS patients >30 kg/m^2^.

#### Sample Size

The pooled MD for plasma IL-6 levels in studies where the total number of participants ≤ 100 was significant (MD = 4.56 pg/ml, *P* < 0.0001); plasma IL-6 levels in those with OSAS was significantly higher than controls. In contrast, there was no significant difference between participants with OSAS and controls in the studies where the total number of participants >100.

#### Apnea–Hypopnea Index

Compared to controls, for participants with OSAS, the plasma IL-6 levels were significantly higher in studies where mean AHI of OSAS participants >30 events/h (MD = 5.87 pg/ml, *P* < 0.00001) but lower in studies where the AHI ≤ 30 events/h (MD = 0.43 pg/ml, *P* = 0.01). The MD in plasma IL-6 levels in studies where mean AHI of OSAS participants >30 events/h was 13.6 times greater than in studies where this mean AHI ≤ 30 events/h.

### Serum IL-6 Levels in Adult Caucasians

#### Body Mass Index

In a further subgroup analysis, the pooled MD comparing serum and plasma IL-6 levels in OSAS adult Caucasians and controls was investigated (Table 3). The pooled MDs in those studies with OSAS participants with a mean BMI > 30 kg/m^2^ (MD = 0.35 pg/ml, *P* = 0.008) and those where the BMI ≤ 30 kg/m^2^ (MD = 2.54 pg/ml, *P* = 0.002) were significant when compared to controls' serum IL-6 levels. This pattern was independent of controls' BMI, whether higher or lower than 30 kg/m^2^. Thus, serum IL-6 level in studies where OSAS participants and controls had a BMI ≤ 30 kg/m^2^ was 7.3 times higher than serum IL-6 level in studies where OSAS participants and controls had a BMI > 30 kg/m^2^.

**Table 3:** Subgroup analysis for serum and plasma levels of inteleukin-6 in adult Caucasians.

**Subgroup analysis of serum level (*n*)**	**MD (95% CI), *P*-value, *I*^**2**^ (%), *P*_**h**_**	**Subgroup analysis of plasma level (*n*)**	**MD (95% CI), *P*-value, *I*^**2**^ (%), *P*_**h**_**
Overall (16)	**0.51 (0.29, 0.73)**, **<0.00001, 97**, **<0.00001**	Overall (10)	0.75 (−0.06, 1.57), 0.07, 98, <0.00001
Mean BMI of OSAS patients, kg/m^2^		Mean BMI of OSAS patients, kg/m^2^	
>30 (11)	**0.35 (0.09, 0.62), 0.008, 87**, **<0.00001**	>30 (5)	0.46 (0.01, 0.91), 0.05, 92, <0.00001
≤ 30 (3)	**2.54 (0.90, 4.19), 0.002, 92**, **<0.00001**	≤ 30 (5)	**0.89 (0.27, 1.50), 0.005, 96**, **<0.00001**
Mean BMI of controls, kg/m^2^		Mean BMI of controls, kg/m^2^	
>30 (11)	**0.35 (0.09, 0.62), 0.008, 87**, **<0.00001**	>30 (3)	2.26 (−5.67, 10.20), 0.58, 99, <0.00001
≤ 30 (3)	**2.54 (0.90, 4.19), 0.002, 92**, **<0.00001**	≤ 30 (7)	**0.71 (0.19, 1.23), 0.007, 95**, **<0.00001**
Total number of participants		Total number of participants	
>100 (3)	**1.81 (0.75, 2.87), 0.0008, 78, 0.01**	>100 (4)	0.56 (−0.28, 1.41), 0.19, 93, <0.00001
≤ 100 (13)	**0.34 (0.11, 0.56), 0.003, 97**, **<0.00001**	≤ 100 (6)	1.14 (−1.72, 4.00), 0.44, 99, <0.00001
Mean AHI of OSAS patients, events/h		Mean AHI of OSAS patients, events/h	
>30 (6)	0.23 (−0.11, 0.57), 0.18, 77, 0.0006	>30 (5)	**1.48 (1.26, 1.70)**, **<0.00001, 26, 0.25**
≤ 30 (6)	**0.61 (0.33, 0.88)**, ** <0.0001, 88**, **<0.0001**	≤ 30 (4)	**0.19 (0.16, 0.23)**, **<0.00001, 24, 0.27**

BMI, body mass index; CI, confidence interval; OSAS, obstructive sleep apnea syndrome; MD, mean difference; P_h_, P_heterogeneity_.

*Bold numbers show statistically significant value (P < 0.05)*.

#### Sample Sizes

With regard to studies of Caucasians, in those studies with more than 100 cases with or without OSAS, the pooled MD for serum IL-6 level was 1.81 pg/ml (*P* = 0.0008). In the studies with ≤ 100 cases with or without OSAS, the pooled MD for serum IL-6 level was 0.34 pg/ml (*P* < 0.003). In other words, serum IL-6 levels in studies with sample sizes >100 were higher than the levels in studies with sample sizes ≤ 100.

#### Apnea–Hypopnea Index

For Caucasian samples, the studies with a mean AHI ≤ 30 events/h in participants with OSAS showed that the pooled MD of serum IL-6 levels was 0.61 pg/mL (*P* < 0.0001). However, serum IL-6 levels did not differ between OSAS participants and controls where AHI was >30 events/h.

### Plasma IL-6 Levels in Adult Caucasians

#### Body Mass Index

The pooled MD was significant for plasma IL-6 levels in studies with Caucasian participants with OSAS and with a mean BMI ≤ 30 kg/m^2^ (MD = 0.89 pg/ml, *P* = 0.005) were compared with controls, but there was no significant difference in those studies where the OSAS participants had a mean BMI > 30 kg/m^2^.

#### Sample Size

The pooled MD of plasma IL-6 levels was independent of sample size (samples smaller or larger than 100 per group).

#### Apnea–Hypopnea Index

The pooled MD for plasma IL-6 levels was significantly higher (7.8 times) in Caucasians with OSAS and a mean AHI > 30 events/h (MD = 1.48 pg/ml; *P* = 0.002) when compared to those with OSAS and a mean AHI ≤ 30 events/h (MD = 0.19 pg/ml; *P* < 0.00001).

### Serum Levels of IL-6 in Adult Asian Participants With and Without OSAS

A subgroup analysis for serum IL-6 levels in adult Asians is reported in Table 4. There were significant differences between individuals with OSAS and controls for those studies reporting for the OSAS group a mean BMI ≤ 30 kg/m^2^ (MD = 11.52 pg/ml, *P* < 0.00001), a mean BMI of controls >30 kg/m^2^ (MD = 4.03 pg/ml, *P* < 0.00001), a mean BMI of controls ≤ 30 kg/m^2^ (MD = 12.30 pg/ml, *P* < 0.00001), a total sample size ≤ 100 (MD = 4.85 pg/ml, *P* < 0.00001), and mean AHI of OSAS individuals >30 events/h (MD = 18.16 pg/ml, *P* < 0.0001).

**Table 4:** Subgroup analysis for serum level of inteleukin-6 in adult Asians.

**Subgroup analysis of serum level (*n*)**	**MD (95% CI), *P*-value, *I*^**2**^ (%), *P*_**h**_**
Overall (14)	**11.52 (10.20, 12.83)**, **<0.00001, 100**, **<0.00001**
**Mean BMI of OSAS patients, kg/m**^**2**^	
>30 (0)	NA
≤ 30 (14)	**11.52 (10.20, 12.83)**, **<0.00001, 100**, **<0.00001**
**Mean BMI of controls, kg/m**^**2**^	
>30 (1)	**4.03 (2.38, 5.68)**, **<0.00001**
≤ 30 (13)	**12.30 (10.92, 13.68)**, **<0.00001, 100**, **<0.00001**
**Total number of participants**	
>100 (5)	15.86 (−11.58, 43.30), 0.26, 100, <0.00001
≤ 100 (9)	**4.85 (3.96, 5.74)**, **<0.00001, 99**, **<0.00001**
**Mean AHI of OSAS patients, events/h**	
>30 (7)	**18.16 (10.17, 26.15)**, ** <0.0001, 100**, **<0.00001**
≤ 30 (0)	NA

BMI, body mass index; CI, confidence interval; OSAS, obstructive sleep apnea syndrome; MD, mean difference; NA, not available; P_h_, P_heterogeneity_.

*Bold numbers show statistically significant value (P < 0.05)*.

### Mean BMI of OSAS Patients >30 kg/m^2^

Table 5 reports the subgroup analysis for serum and plasma IL-6 levels in adults where the mean BMI of those with OSAS > 30 kg/m^2^.

**Table 5:** Subgroup analysis for serum and plasma levels of inteleukin-6 in adults where mean BMI of OSAS patients >30 kg/m^2^.

**Subgroup analysis of serum level (*n*)**	**MD (95% CI), *P*-value, *I*^**2**^ (%), *P*_**h**_**	**Subgroup analysis of plasma level (*n*)**	**MD (95% CI), *P*-value, *I*^**2**^ (%), *P*_**h**_**
Overall (15)	**1.07 (0.63, 1.50)**, **<0.00001, 97**, **<0.00001**	Overall (8)	0.96 (−1.16, 3.07), 0.38, 98, <0.00001
Mean BMI of controls, kg/m^2^		Mean BMI of controls, kg/m^2^	
>30 (11)	**0.35 (0.09, 0.62), 0.008, 87**, **<0.00001**	>30 (4)	1.69 (−4.61, 8.00), 0.60, 99, <0.00001
≤ 30 (4)	**3.16 (1.00, 5.32), 0.004, 95**, **<0.00001**	≤ 30 (4)	**0.50 (0.16, 0.85), 0.004, 33, 0.21**
Total number of participants		Total number of participants	
>100 (2)	2.06 (−0.19, 4.31), 0.07, 89, 0.003	>100 (2)	0.31 (−0.09, 0.70), 0.13, 0, 0.79
≤ 100 (13)	**0.93 (0.47, 1.39)**, **<0.0001, 97**, **<0.00001**	≤ 100 (6)	1.41 (−2.29, 5.11), 0.45, 99, <0.00001
Mean AHI of OSAS patients, events/h		Mean AHI of OSAS patients, events/h	
>30 (6)	0.23 (−0.11, 0.57), 0.18, 77, 0.0006	>30 (4)	**1.38 (0.96, 1.79)**, **<0.00001, 0, 0.53**
≤ 30 (5)	0.89 (−0.28, 2.05), 0.14, 62, 0.03	≤ 30 (3)	**0.38 (0.05, 0.71), 0.02, 0, 0.78**

BMI, body mass index; CI, confidence interval; OSAS, obstructive sleep apnea syndrome; MD, mean difference; P_h_, P_heterogeneity_.

*Bold numbers show statistically significant value (P < 0.05)*.

In participants with OSAS, serum IL-6 levels were significantly higher when the mean BMI of controls was ≤ 30 (MD = 3.16 pg/ml, *P* = 0.004); likewise, serum IL-6 levels were lower where the mean BMI of controls was >30 (MD = 0.35 pg/ml, *P* = 0.008), and where the total sample size was ≤ 100 (MD = 0.93 pg/ml, *P* < 0.0001).

Furthermore, among those with OSAS, mean plasma IL-6 levels were significantly higher in studies where the mean BMI of controls ≤ 30 kg/m^2^ (MD = 0.50 pg/ml, *P* = 0.004), in studies where the mean AHI of individuals with OSAS > 30 events/h (MD = 1.38 pg/ml, *P* < 0.00001), and those where the mean AHI of individuals with OSAS ≤ 30 events/h (MD = 0.38 pg/ml, *P* = 0.02).

### Mean BMI of OSAS Patients ≤ 30 kg/m^2^

The subgroup analysis for serum and plasma levels of IL-6 of adults where the mean BMI of individuals with OSAS ≤ 30 kg/m^2^ is reported in Table 6.

**Table 6:** Subgroup analysis for serum and plasma levels of inteleukin-6 in adults where mean BMI of OSAS patients ≤ 30 kg/m^2^.

**Subgroup analysis of serum level (*n*)**	**MD (95% CI), *P*-value, *I*^**2**^ (%), *P*_**h**_**	**Subgroup analysis of plasma level (*n*)**	**MD (95% CI), *P*-value, *I*^**2**^ (%), *P*_**h**_**
Overall (19)	**10.59 (8.64, 12.53)**, **<0.00001, 100**, **<0.00001**	Overall (11)	**4.20 (2.95, 5.46)**, **<0.00001, 99**, **<0.00001**
Mean BMI of controls, kg/m^2^		Mean BMI of controls, kg/m^2^	
>30 (1)	**4.03 (2.38, 5.68)**, **<0.00001**	>30 (0)	NA
≤ 30 (18)	**11.02 (9.01, 13.04)**, **<0.00001, 100**, **<0.00001**	≤ 30 (11)	**4.10 (2.86, 5.34)**, **<0.00001, 99**, **<0.00001**
Total number of participants		Total number of participants	
>100 (7)	**11.92 (2.31, 21.52), 0.02, 100**, **<0.00001**	>100 (3)	0.49 (−0.08, 1.06), 0.09, 96, <0.00001
≤ 100 (12)	**8.59 (6.89, 10.29)**, **<0.00001, 99**, **<0.00001**	≤ 100 (8)	**7.06 (3.68, 10.44)**, **<0.0001, 99**, **<0.00001**
Mean AHI of OSAS patients, events/h		Mean AHI of OSAS patients, events/h	
>30 (7)	**18.16 (10.17, 26.15)**, **<0.00001, 100**, **<0.00001**	>30 (6)	**8.48 (4.75, 12.21)**, **<0.00001, 99**, **<0.00001**
≤ 30 (2)	1.05 (−0.16, 2.27), 0.09, 71, 0.06	≤ 30 (4)	0.16 (−0.16, 0.48), 0.32, 68, 0.02

BMI, body mass index; CI, confidence interval; OSAS, obstructive sleep apnea syndrome; MD, mean difference; NA, not available; P_h_, P_heterogeneity_.

*Bold numbers for statistically significant values (P < 0.05)*.

There was significantly higher levels of serum IL-6 in individuals with OSAS, both when controls had BMIs lower than 30 kg/m^2^ (MD = 11.02 pg/ml, *P* < 0.00001) and when this was higher than 30 kg/m^2^ (MD = 4.03 pg/ml, *P* < 0.00001), when overall sample size was both <100 (MD = 8.59 pg/ml, *P* < 0.00001) and >100 (MD = 11.92 pg/ml, *P* = 0.02), and when the mean AHI of individuals with OSAS was higher than 30 (MD = 18.16 pg/ml, *P* < 0.00001).

Under the following conditions, plasma levels of IL-6 were significantly higher: the mean BMI of controls was ≤ 30 kg/m^2^ (MD = 4.10 pg/ml, *P* < 0.00001), total sample size was ≤ 100 (MD = 7.06 pg/ml, *P* < 0.0001), and mean AHI of individuals with OSAS was ≤ 30 events/h (MD = 8.48 pg/ml, *P* < 0.00001).

### Meta-Regression

The results of meta-regression showed that with greater age, serum IL-6 levels were significantly higher. The publication year, the mean BMI, the mean AHI, and number of participants had no independent significant effects on serum or plasma IL-6 levels (Table 7).

**Table 7:** Meta-regression analysis of variables predicting serum and plasma levels of inteleukin-6 comparing obstructive sleep apnea syndrome with controls.

**Year of publication**	***R***	**Adjusted *R*^**2**^**	***P***	**Mean age of OSAS patients**	***R***	**Adjusted *R*^**2**^**	***P***	**Mean age of controls**	***R***	**Adjusted *R*^**2**^**	***P***
Serum	0.001	−0.029	0.995	Serum	0.479	0.207	**0.03**	Serum	0.385	0.123	**0.022**
Plasma	0.124	−0.034	0.582	Plasma	0.377	0.092	0.112	Plasma	0.204	−0.018	0.417
Mean BMI of OSAS patients	*R*	Adjusted *R*^2^	*P*	Mean BMI of controls	*R*	Adjusted *R*^2^	*P*	Mean AHI of OSAS patients	*R*	Adjusted *R*^2^	*P*
Serum	0.119	−0.017	0.504	Serum	0.089	−0.023	0.615	Serum	0.188	−0.011	0.391
Plasma	0.143	−0.031	0.538	Plasma	0.168	−0.032	0.504	Plasma	0.169	−0.036	0.517
Number of participants	*R*	Adjusted *R*^2^	*P*								
Serum	0.062	−0.025	0.717								
Plasma	0.159	−0.029	0.504								

*R, correlation coefficient. Bold numbers for statistically significant values (P < 0.05)*.

### Quality Assessment

The quality scores of the studies included in the meta-analysis are reported in Table 8.

**Table 8:** Quality assessment scores of the studies involved in the meta-analysis.

**The first author, year**	**Selection**	**Comparability**	**Exposure**	**Total points**
**ADULTS**				
Vgontzas, 1997 (26)	***	–	***	6
Huiguo, 2000 (27)	***	**	***	8
Roytblat, 2000 (28)	***	*	***	7
Teramoto, 2003 (29)	**	–	***	5
Yokoe, 2003 (30)	***	**	***	8
Ciftci, 2004 (12)	***	**	***	8
Imagawa, 2004 (31)	***	*	***	7
Ryan, 2006 (32)	***	**	***	8
de la Peña Bravo, 2007 (34)	***	**	***	8
Harsch, 2007 (35)	***	**	***	8
Arias, 2008 (36)	***	**	***	8
Constantinidis, 2008 (37)	***	*	***	7
Li, 2008 (40)	****	**	***	9
Takahashi, 2008 (41)	***	**	***	8
Tomiyama, 2008 (42)	***	**	***	8
Vgontzas, 2008 (43)	***	**	***	8
Yamamoto, 2008 (44)	***	**	***	8
Carneiro, 2009 (45)	***	**	***	8
Li, 2009 (46)	***	**	***	8
Thomopoulos, 2009 (47)	***	**	***	8
Sahlman, 2010 (48)	***	**	***	8
Steiropoulos, 2010 (49)	***	**	***	8
Ye, 2010 (50)	****	**	***	9
Liu, 2011(51)	***	**	***	8
Fornadi, 2012 (52)	***	**	***	8
Medeiros, 2012 (53)	***	**	***	8
Qian, 2012 (54)	***	**	***	8
Ye, 2012 (55)	***	**	***	8
Hargens, 2013 (56)	***	**	***	8
Kurt, 2013 (57)	***	*	***	7
Yang, 2013 (58)	***	**	***	8
Ciccone, 2014 (59)	***	**	***	8
Kritikou, 2014 (61)	***	**	***	8
Unuvar Dogan, 2014 (62)	***	**	***	8
De, 2015 (63)	***	**	***	8
Gaines, 2015 (64)	***	**	***	8
Hui, 2015 (65)	**	-	***	5
Ulasli, 2015 (67)	***	**	***	8
Thunstrom, 2015 (66)	****	**	***	9
Dogan, 2016 (68)	***	**	***	8
Nizam, 2016 (70)	***	**	***	8
Vicente, 2016 (71)	***	**	***	8
Hirotsu, 2017 (72)	****	**	***	9
Kong, 2017 (73)	***	**	***	8
Thorn, 2017 (74)	***	**	***	8
Weingarten, 2017 (76)	***	**	***	8
Zhang, 2017 (77)	***	*	***	7
Al-Terki, 2018 (78)	***	**	***	8
Bhatt, 2018 (79)	***	**	***	8
Bozic, 2018 (80)	***	**	***	8
Kong, 2018 (81)	***	**	***	8
Lu, 2018 (82)	***	**	***	8
Motamedi, 2018 (83)	****	**	***	9
Sundbom, 2018 (84)	****	**	***	9
Bhatt, 2019 (85)	***	*	***	7
Tang, 2019 (86)	***	**	***	8
Galati, 2020 (87)	****	**	***	8
**CHILDREN**				
Tam, 2006 (33)	****	**	***	9
Gozal, 2008 (38)	****	**	***	9
Li, 2008 (39)	***	*	***	7
Gileles-Hillel, 2014 (60)	***	**	***	8
Huang, 2016 (69)	***	*	***	7
Van Eyck, 2017 (75)	***	*	***	7

*Each asterisk denotes 1 point*.

### Sensitivity Analysis

The “cumulative analysis” and the “one study removed” as two sensitivity analyses confirmed the stability of the results. In addition, excluding studies with outlier data did not affect the pooled analysis of serum (MD = 2.89 pg/ml, *P* < 0.00001) or plasma (MD = 2.78 pg/ml, *P* < 0.00001) IL-6 levels (Table 9).

**Table 9:** Sensitivity analysis on the results of serum and plasma levels in adults.

**First author, year**	**Sample**	**Reason for removing**	**MD (95% CI)**	***Z***	***P* value**	***I*^**2**^**	***P*_**h**_**
Hargens, 2013	Serum	Outlier data	2.89 (2.54, 3.24)	16.04	<0.00001	99%	<0.00001
Harsch, 2007	Plasma	Outlier data	2.78 (1.91, 3.66)	6.24	<0.00001	99%	<0.00001

### Publication Bias

**Figure 6:**
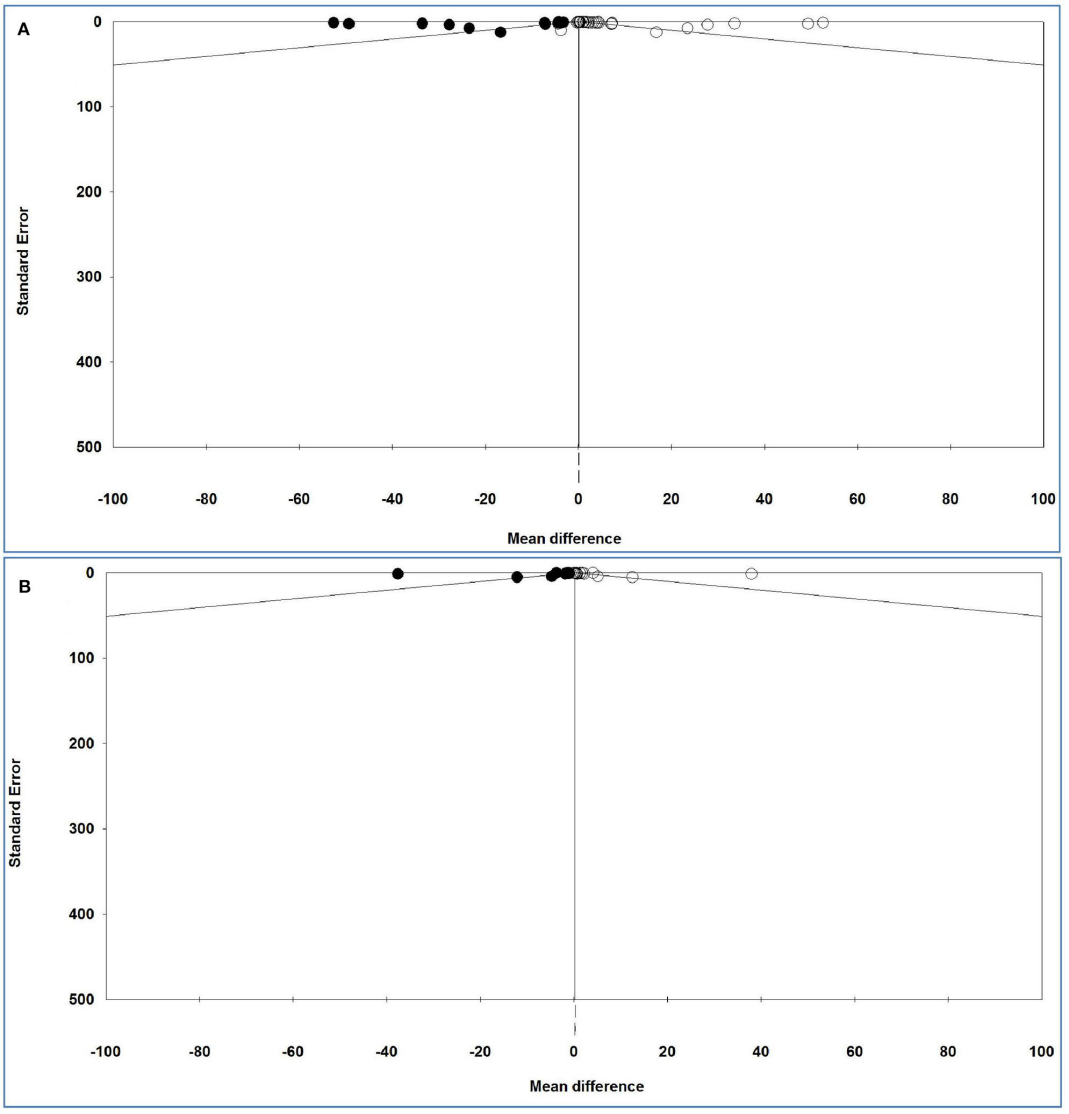
Funnel plot of analysis of interleukin-6 levels in adult participants on **(A)** serum and **(B)** plasma in adult participants. Open circles represent observed studies. Black circles represent imputed studies. Open diamonds represent the pooled effects from the original studies. Black diamonds represent the pooled effects incorporating the imputed studies.

The funnel plots of the analysis of serum and plasma IL-6 levels are shown in Figure 6, and Table 10 gives the results of the trim-and-fill method on bias.

**Table 10:** The results of trim-and-fill method.

**Sample**	**Value**	**Studies trimmed**	**Fixed effects**			**Random effects**			***Q* value**
			**Point estimate**	**Lower limit**	**Upper limit**	**Point estimate**	**Lower limit**	**Upper limit**	
Serum	Observed	–	0.02403	0.01818	0.02988	2.41151	2.07576	2.74726	4,869.43934
	Adjusted	14	0.02287	0.01702	0.02872	0.45786	0.08567	0.83005	7,858.53130
Plasma	Observed	–	0.25196	0.22049	0.28343	2.94160	1.87192	4.01128	1,640.40890
	Adjusted	10	0.20025	0.116914	0.23136	0.28995	−0.73377	1.31366	3,295.78126

For serum and plasma IL-6 levels, Egger's test (*P* = 0.00044 and *P* = 0.01445, respectively) revealed a publication bias, but Begg's test (*P* = 0.44811 and *P* = 0.55922, respectively) indicated no bias either between or across the studies.

For serum IL-6 levels and 14 imputed studies, under the fixed-effects model, the point estimate and pseudo 95% CI for the combined studies is 0.024 (0.018, 0.030); using the trim–fill method, the imputed point estimate is 0.023 (0.017, 0.029). In addition, under the random-effects model, the point estimate and 95% CI for the combined studies is 2.411 (2.076, 2.747); using the trim–fill method, the imputed point estimate is 0.458 (0.086, 0.830).

For plasma IL-6 levels and 10 imputed studies, under the fixed-effects model, the point estimate and 95% CI for the combined studies is 0.252 (0.220, 0.283), and using trim–fill method, the imputed point estimate is 0.200 (0.117, 0.231). In addition, under the random-effects model, the point estimate and pseudo 95% CI for the combined studies is 2.941 (1.872, 4.011); using the trim–fill method, the imputed point estimate is 0.290 (−0.734, 1.314).

The overall effect sizes for serum and plasma IL-6 levels reported in the forest plot appears valid, with a trivial publication bias effect based on a fixed-effects model, because the observed estimates were similar to the adjusted estimates. In contrast, the overall effect sizes on serum and plasma IL-6 levels reported in the forest plot appeared invalid, with a significant publication bias effect based on random-effects model, because the observed estimates were substantially different to the adjusted estimates.

## Discussion

The present meta-analysis with 63 studies evaluated serum IL-6 levels (37 studies of adults and three of children) and plasma IL-6 levels (20 studies of adults and three of children) comparing individuals with OSAS to controls. The analysis showed that plasma and serum IL-6 levels of adults with OSAS were significantly higher than the corresponding levels of control. For children with OSAS, plasma IL-6 levels but not serum IL-6 levels were significantly higher than those of controls. The present conclusions have clinical importance because an elevated IL-6 level can be a risk factor for the development of cardiovascular diseases in adults with OSAS (88). These conclusions also have practical importance because, in addition to routine checks on sleep and sleep-disordered breathing, both children and adults with OSAS need a thorough monitoring of their immune systems.

One study (89) has shown that OSAS can alter the secretory levels of several hormones. However, vascular and systemic inflammation is the main pathogenesis of OSAS-related cardiac metabolic processes via activation of inflammatory pathways (90). Nadeem et al. (9) concluded, in their meta-analysis of 14 studies, that adults with OSAS had higher serum levels of IL-6 than healthy controls. Zhong et al. (16) similarly concluded from their meta-analysis that both children (four studies) and adults with OSAS (31 studies) had significantly higher serum IL-6 levels than controls. Although the exact mechanism of the OSAS-induced impact on levels of IL-6 is not known, both sleep deprivation and hypoxemia are believed to be important causative factors (32).

IL-6 is the cytokine with the most clearly documented effects on nonimmunological tissues and has been termed a pleiotropic or “endocrine” cytokine (91). In healthy individuals, inflammatory cytokines such as IL-6 are involved in the regulation of physiological sleep associated with the circadian secretion pattern (92). A good night's sleep and a good sense of well-being on the following day can be associated with reduced IL-6 secretion (93), whereas increased secretion of this cytokine can cause excessive daytime sleepiness (EDS) and fatigue (26, 94). In this respect, we note that EDS is a principal complaint of patients with sleep disorders (95) and is one of the most important physiological consequences of OSAS (96). In a similar vein, a meta-analysis of studies measuring the IL-6 level of patients with major depression found that these levels were significantly higher than in controls (97).

Furthermore, previous studies (26, 94) have confirmed that serum IL-6 levels are positively correlated with higher BMI scores as a proxy for obesity. Roytblat et al. (28) evaluated serum IL-6 levels of individuals with obesity, individuals with OSAS, and healthy controls. They found that IL-6 levels were 34-fold higher in individuals with obesity hypoventilation than in controls, and 8-fold higher in individuals with OSAS. In addition, serum cytokine levels were higher in all individuals with obesity. To explain these results, it appears that oxidative stress, inflammation, and sympathetic activation as proxies for pathophysiological mechanisms known to be present in individuals with OSAS also occur in individuals with obesity (98). The correlations between the levels of cytokines, apnea, and obesity are unclear (63). In our meta-analysis, based on subgroup analyses, the serum and plasma IL-6 levels in obese participants with OSAS were very much lower than in nonobese OSAS participants. However, some studies (26, 63, 68, 99) have reported a positive and significant correlation between BMI and IL-6 levels in OSAS cases, although other studies (12, 51) failed to find any significant correlation. Therefore, the higher MD of IL-6 levels in nonobese OSAS samples found in the present analysis may possibly indicate that BMI by itself does not affect IL-6 levels in OSAS patients but that there are other confounding factors influencing the serum and plasma IL-6 levels.

Next, several studies (26, 30, 66, 83) have shown that individuals with severe OSAS have higher mean plasma/serum IL-6 levels than either those with mild OSAS or controls. Furthermore, higher serum IL-6 levels have been found to be significantly correlated with higher AHI in individuals with OSAS (*r* = 0.33, *P* = 0.03) (12). Our subgroup analysis similarly showed that serum and plasma levels of IL-6 of individuals with an AHI > 30 events/h was higher than the levels of individuals with an AHI ≤ 30 events/h; this was the case in particular for participants with a mean BMI ≤ 30 kg/m^2^ and of Asian background. It follows that Asians with AHI > 30 events/h and BMI ≤ 30 kg/m^2^ are at the greatest risk of having high serum IL-6 levels. The data available from the studies included in our analysis are insufficient to clarify the meaning of the patterns of results observed and in particular cannot shed any direct light on the underlying physiological mechanisms. It follows that future studies should investigate the extent to which ethnicity and its genetic make-up might be associated with vulnerability.

Despite the novelty of the findings, the following limitations should be considered. First, in none of the studies were results adjusted to reflect possible confounding factors such as obesity, smoking, or alcohol consumption. Second, the results of the funnel plots showed a publication bias across the studies; it follows that a systematic bias in data presentation cannot be ruled out. Third, the studies with small sample sizes (<100) had insufficient power to detect associations. Fourth, there was a high level of heterogeneity among studies with respect to some analyses. Fifth, studies reported different cutoff values for AHI, making comparisons between the studies difficult. Sixth, in some studies, level of IL-6 was treated as a secondary outcome.

In contrast, the strengths of the meta-analysis were as follows. First, the meta-regression revealed that age could have a confounding effect on serum results. Second, there were sufficient studies to allow for subgroup analysis. Third, sensitivity analysis confirmed the stability of results. Fourth, studies published in languages other than English were included in the meta-analysis.

## Conclusions

The results of the present meta-analysis showed higher levels of IL-6 in individuals with OSAS to be related to the severity of the disease. Furthermore, age has an impact on the associations between OSAS and IL-6 levels. Future studies might investigate the extent to which interventions to address OSAS (e.g., using CPAP devices) impact positively on IL-6 levels and possibly also on weight regulation.

## Practice Points

- Both children and adults with OSAS need careful monitoring of their immune system, as plasma and serum IL-6 concentrations are higher than in healthy counterparts.
- This statement holds particularly true for older individuals with OSAS and with higher BMI scores.
- Individuals with OSAS are at particular risk of suffering from other illnesses related to an impaired immune system.

## Research Agenda

- In individuals with OSAS and receiving CPAP treatment, IL-6 concentrations should be assessed before and after the CPAP treatment as an indicator of a normalized psychophysiological recovery.
- In individuals with OSAS and tonsillectomy, IL-6 concentrations should be assessed before and after the surgical intervention as an indicator of a normalized psychophysiological recovery.
- In individuals with OSAS and receiving appropriate treatment, IL-6 concentrations should be associated with possible changes in academic performance and social behavior.
- In overweight individuals with OSAS, assessment of the family system (overweight/obese parents or siblings) might explain why OSAS could persist in an undetected fashion.
- Retrospective chart records should calculate the extent to which OSAS and a deteriorated immune system might be associated with DALYS (disability-adjusted life years).

## Data Availability Statement

All datasets generated for this study are included in the article.

## Author Contributions

All authors contributed to the article and approved the submitted version.

## Conflict of Interest

The authors declare that the research was conducted in the absence of any commercial or financial relationships that could be construed as a potential conflict of interest.

## Abbreviations

AHI: apnea–hypopnea index
BMI: body mass index
CI: confidence interval
CPAP: continuous positive air pressure
DALYS: disability-adjusted life years
EDS: excessive daytime sleepiness
IL-6: interleukin-6
MD: mean difference
NFκB: nuclear factor κB
NOS: Newcastle–Ottawa Scale
OSAS: obstructive sleep apnea syndrome
PRISMA: Preferred Reporting Items for Systematic Reviews and Meta-analyses
SD: standard deviation.
